# Decreased incidence of Kawasaki disease in South Korea during the SARS-CoV-2 pandemic

**DOI:** 10.3389/fped.2024.1307931

**Published:** 2024-04-03

**Authors:** Kyung Jin Oh, Sang-Yun Lee

**Affiliations:** ^1^ Department of Pediatrics, Seoul Metropolitan Government-Seoul National University Boramae Medical Center, Seoul, Republic of Korea; ^2^ Department of Pediatrics, Seoul National University Children's Hospital, Seoul National University College of Medicine, Seoul, Republic of Korea

**Keywords:** Kawasaki disease, coronavirus pandemic, mucocutaneous lymph node syndrome, etiology, big data

## Abstract

**Purpose:**

Analyzing Kawasaki disease epidemiology during the SARS-CoV-2 pandemic in South Korea using 2012–2020 National Health Insurance Service data.

**Methods:**

The incidence of Kawasaki disease for 2012–2020 was investigated to identify changes in incidence after the start of the pandemic. National Health Insurance Service data from the Republic of Korea were used. Kawasaki disease was defined based on the International Statistical Classification of Diseases and Related Health Problems, the Tenth Revision diagnostic code (M30.3), and the intravenous immunoglobulin prescription code. Prescription history was collected for the following medications: intravenous immunoglobulin, aspirin, corticosteroids, tumor necrosis factor-α antagonist, clopidogrel, and anticoagulation drugs.

**Results:**

The Kawasaki disease incidence per 100,000 individuals younger than 5 years was 238.9, 230.0, and 141.2 in 2018, 2019, and 2020, respectively. Regarding the incidence from 2012 to 2020, it was the highest in 2018 and decreased to 141.2 (*p* < 0.001) in 2020, after the start of the pandemic. In 2020, 28.3% of all patients with KD were infants, a percentage significantly higher than that of the previous year (*p* < 0.001). There was biphasic seasonality in the monthly Kawasaki disease incidence. The Kawasaki disease incidence was the highest in winter followed by that in early summer.

**Conclusion:**

After the start of the pandemic, the Kawasaki disease incidence decreased, and the percentage of patients with Kawasaki disease aged <1 year increased. These findings provide support for the hypothesis suggesting an infectious trigger in Kawasaki disease.

## 1 Introduction

Kawasaki disease (KD), the most common acquired heart disease in children, is characterized by systemic vasculitis that primarily affects medium-sized blood vessels. It can cause rare cardiovascular complications such as coronary artery aneurysms in approximately 25% of patients if excessive inflammatory reactions are not treated appropriately in the acute phase (1). Hence, timely diagnosis and treatment are very important because KD can cause serious complications such as myocardial ischemia in children (1, 2). Although KD was first reported in 1967, it is still diagnosed based on the clinical symptoms established by Dr. Kawasaki (1, 3). Notably, the lack of characteristic manifestations can make the detection of KD difficult, sometimes leading to the diagnosis of atypical KD (4). An exploration of challenges associated with examining patients with KD has revealed the intricate nature of achieving a precise diagnosis. This complexity is rooted in the elusive understanding of the exact cause and mechanism of KD. Previous studies have suggested that KD is likely triggered by certain factors, which include infectious agents and environmental elements, in children who are genetically or immunologically predisposed (5, 6).

Before 2019, i.e., before the Coronavirus Disease 2019 (COVID-19) pandemic, KD incidence was consistently increasing in Asian countries such as the Republic of Korea, Japan, and Taiwan (7, 8). In contrast, KD incidence was almost stable without any marked changes in North American and European countries (9). However, this incidence trend significantly declined during the COVID-19 pandemic in most countries (10–12). This decline can be attributed, in part, to the implementation of social distancing during the pandemic, a nonpharmaceutical intervention that contributed to a decrease in the incidence of infectious diseases in pediatric patients (13, 14).

The reduction in KD incidence during the pandemic further substantiates the hypothesis that infections could act as a trigger for KD. The decrease in the number of children with KD owing to environmental changes, such as the restriction of human-to-human contact, which is difficult to create artificially, is tangible. Analyzing the impact of environmental changes on KD incidence can provide important insights into disease mechanisms. Despite two studies on this trend, discrepancies in KD incidence from the same data source suggest a need for refined data collection (15, 16) (Supplementary Appendix Table S1, S2). Our objective was to analyze the data from the Health Insurance Review and Assessment Service (HIRA) spanning from 2012 to 2020, under specific conditions, to accurately depict KD trends during the COVID-19 pandemic and address any inconsistencies.

## 2 Materials and methods

KD incidence from 2012 to 2020 was investigated to identify changes in the incidence after the start of the COVID-19 pandemic. Data from the National Health Insurance Service (NHIS) of the Republic of Korea, a universal health reimbursement insurer that covers approximately 98% of the Korean population, were used. The HIRA database was used as the source for KD data. The data collected included province, age, sex, diagnosis, prescriptions, and procedures covered by the NHIS. KD was defined based on the International Statistical Classification of Diseases and Related Health Problems, the Tenth Revision diagnostic code (M30.3), and the prescription code of intravenous immunoglobulin (IVIG). The data request was made in May 2021; permission for data access was approved by the HIRA in February 2022. The data on the population under 5 years of age and COVID-19 outbreak statistics were obtained from the Korean Statistical Information Service (KOSIS). KOSIS is a national statistical database operated by Statistics Korea, an esteemed organization that maintains an exhaustive repository of reliable and trustworthy demographic data.

Single-dose IVIG-responsive KD is defined as the subsidence of fever without additional IVIG administration after continuous intravenous administration of IVIG at a dose of 2 g/kg for 10–12 h. Prescription history for the following medications (intravenous immunoglobulin, aspirin, corticosteroids, clopidogrel, and anticoagulation drugs) can be found in Supplementary Appendix Table S3.

### 2.1 Ethical approval

This study was approved by the institutional review board and conducted in accordance with the principles of the Declaration of Helsinki (IRB approval number: 2111-070-1271, dated November 12, 2021).

### 2.2 Statistical analysis

Data were analyzed using R version 4.0.0 (R Foundation for Statistical Computing, Vienna, Austria). For descriptive analysis, frequencies and percentages were calculated for categorical variables and means and standard deviations for continuous variables. To determine incidence rates per 100,000, population data for individuals aged <5 years obtained from KOSIS were used as the denominator. Additionally, SPSS for Windows version 20.0 was used to analyze the correlation of incidence rates between KD and COVID-19. The frequency of COVID-19 cases was obtained using national data provided by KOSIS. Our data did not show normality; thus, we used the nonparametric Spearman correlation analysis method.

## 3 Results

**Figure 1:**
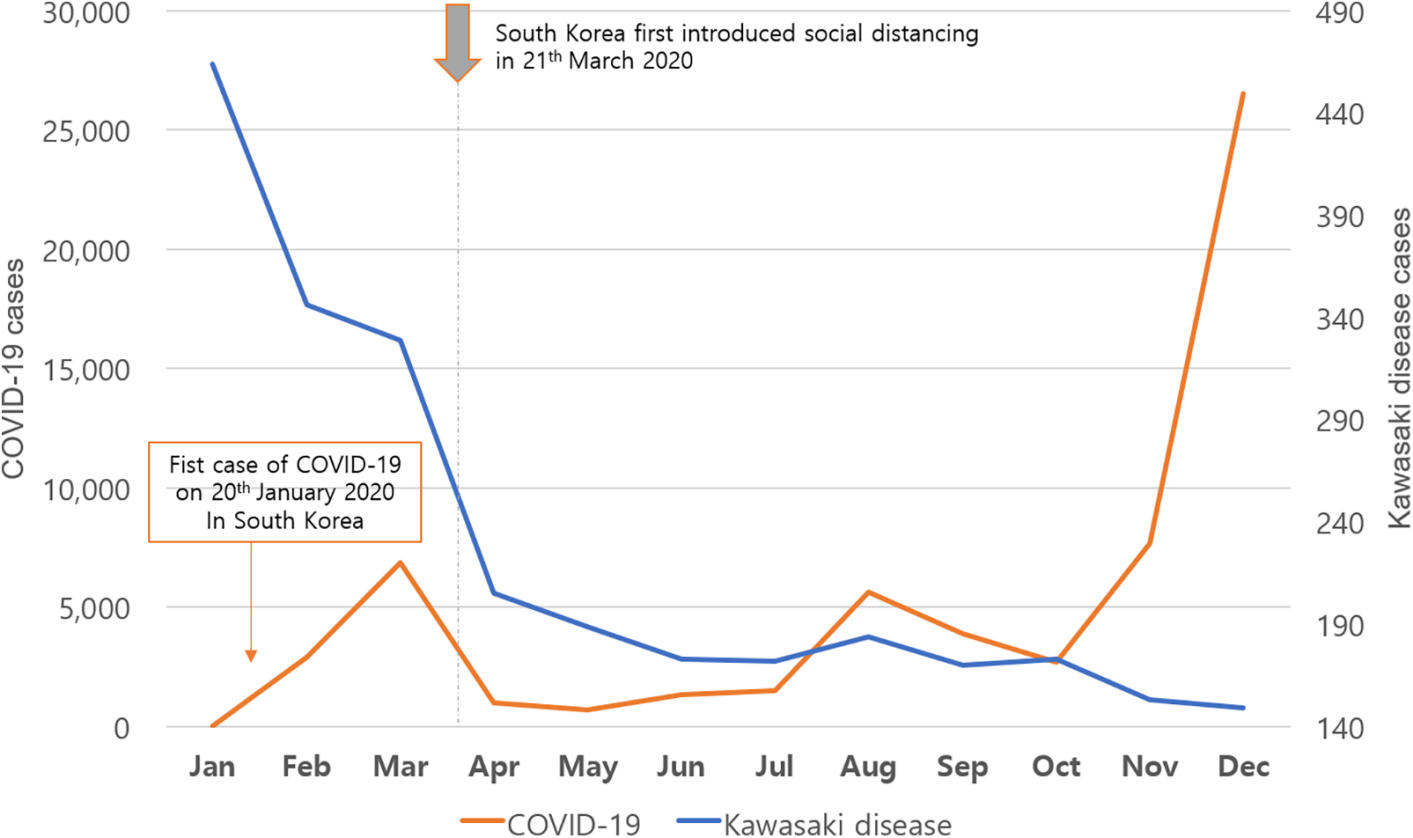
Monthly incidence of Kawasaki disease and coronavirus disease 2019 (COVID-19) during 2020.

**Figure 2:**
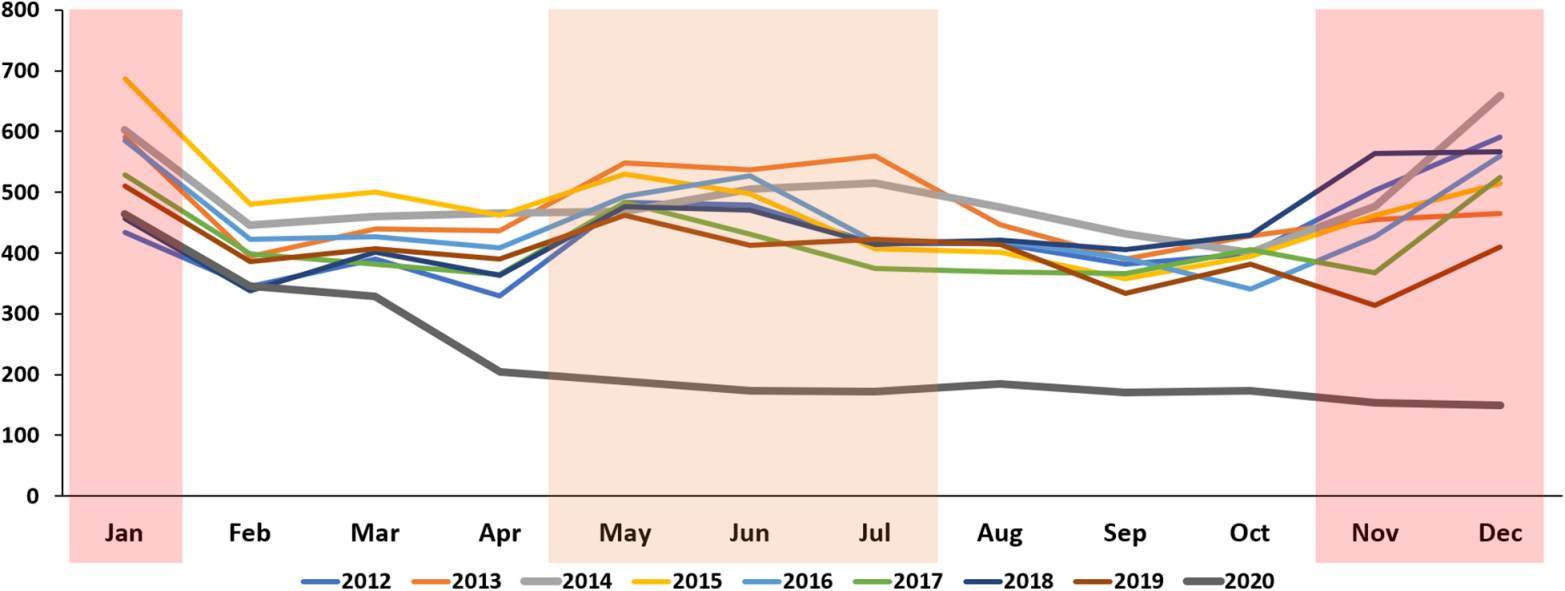
Monthly trends in the incidence of Kawasaki disease per 100,000 individuals younger than 5 years in the Republic of Korea from 2012 to 2020.

More than 45,000 patient data points from the HIRA database were analyzed (Table 1). Approximately 5,000 patients were confirmed every year. The KD incidence per 100,000 individuals younger than 5 years was 238.9, 230, and 141.2 in 2018, 2019, and 2020, respectively. Regarding the incidence of KD from 2012 to 2020, the highest value was observed in 2018. The incidence in 2020, after the start of the COVID-19 pandemic, significantly decreased to 141.2 (*p* < 0.001). KD patients are predominantly male, with the male-to-female patient ratio being 1.32–1.47. The ratio did not differ after the start of the pandemic. Furthermore, the percentage of KD patients aged <1 year reported in 2020 was 28.3%, which was significantly higher than that in the previous year (*p* < 0.001) (Table 1). To investigate the association between KD and COVID-19, our study analyzed the correlation of KD incidence with the emergence of COVID-19 infections during 2020 (Figure 1). The obtained coefficient value was −0.443 (*p* = 0.062), suggesting a negative correlation between KD and COVID-19 cases. The first coronavirus case appeared on January 20, 2020, and the first official social distancing measures were implemented in the country in March, 2 months after the first outbreak (17). Further analysis focused on cases from April through December 2020, which would have been affected by these social distancing measures, revealing a more pronounced correlation coefficient of −0.787 (*p* < 0.05) was observed, indicating a statistically significant negative correlation. The monthly KD incidence showed biphasic seasonality between 2012 and 2019. The incidence was the highest in the winter (December and January) followed by early summer (May–July) (Figure 2). However, in 2020, the monthly incidence remained constant without seasonal changes. This loss of seasonal change has been prominent since February 2020, when social quarantine was implemented due to the start of the pandemic.

**Table 1:** The incidence of Kawasaki disease in individuals younger than 5 years in the Republic of Korea from 2012 to 2020.

		2012	2013	2014	2015	2016	2017	2018	2019	2020
M/F ratio		1.36	1.41	1.38	1.36	1.47	1.35	1.32	1.33	1.41
No. of cases	<1 year	1,255.0	1,215.0	1,262.0	1,272.0	1,212.0	1,132.0	1,191.0	965.0	765.0
	1 to <5 years	3,434.0	3,962.0	4,181.0	3,950.0	3,712.0	3,437.0	3,587.0	3,362.0	1,666.0
	<5 years	4,689.0	5,177.0	5,443.0	5,222.0	4,924.0	4,569.0	4,778.0	4,327.0	2,431.0
	All ages	5,363.0	5,691.0	5,907.0	5,693.0	5,422.0	4,995.0	5,305.0	4,843.0	2,707.0
Infantile KD percentage (<1 year/no. of All ages KD case) (%)		23.4	21.3	21.4	22.3	22.4	22.7	22.5	19.9	28.3
KD incidence of 100,000 population (<5 years)		201.7	224.0	236.9	231.2	222.0	217.3	238.9	230.0	141.2
Total population of South Korea (<1 years)		473,981.0	464,763.0	436,868.0	441,685.0	429,094.0	385,224.0	348,253.0	321,317.0	292,294.0
Total population of South Korea (<5 years)		2,324,347.0	2,310,730.5	2,297,243.5	2,258,670.0	2,218,378.0	2,102,959.0	2,000,217.0	1,881,548.0	1,722,081.0

During the clinical course, the duration of hospitalization ranged from 6.3 to 6.5 days and the percentage of KD refractory to single-dose IVIG was 8.9–15.4%. These values did not change after the start of the pandemic. Although steroid use did not change markedly between 2012 and 2020, the use of clopidogrel, tumor necrosis factor-α antagonists, and anticoagulation drugs showed increasing trends (Table 2). The increased use of these drugs was estimated to be due to NHIS reimbursement.

**Table 2:** The clinical features of Kawasaki disease in individuals younger than 5 years in the Republic of Korea from 2012 to 2020.

Year		2012	2013	2014	2015	2016	2017	2018	2019	2020
Total no. of cases		5,363	5,691	5,907	5,693	5,422	4,995	5,305	4,843	2,707
Duration of hospitalization	(Days)	6.4 ± 3.2	6.4 ± 3.5	6.4 ± 2.7	6.4 ± 3.5	6.3 ± 3.1	6.3 ± 2.9	6.3 ± 2.6	6.3 ± 2.8	6.5 ± 3.9
KD refractory to single-dose IVIG	(No. of cases)	460	523	628	632	585	644	747	745	305
	(%)	8.90	9.19	10.63	11.10	10.79	12.89	14.08	15.38	11.26
Methylprednisolone	(No. of cases)	229	293	390	531	471	555	557	690	404
Infliximab		1	2	3	6	5	16	11	9	28
Clopidogrel		40	74	93	109	129	152	306	312	198
Anticoagulation agents		20	22	22	30	36	49	102	106	100

**Figure 3:**
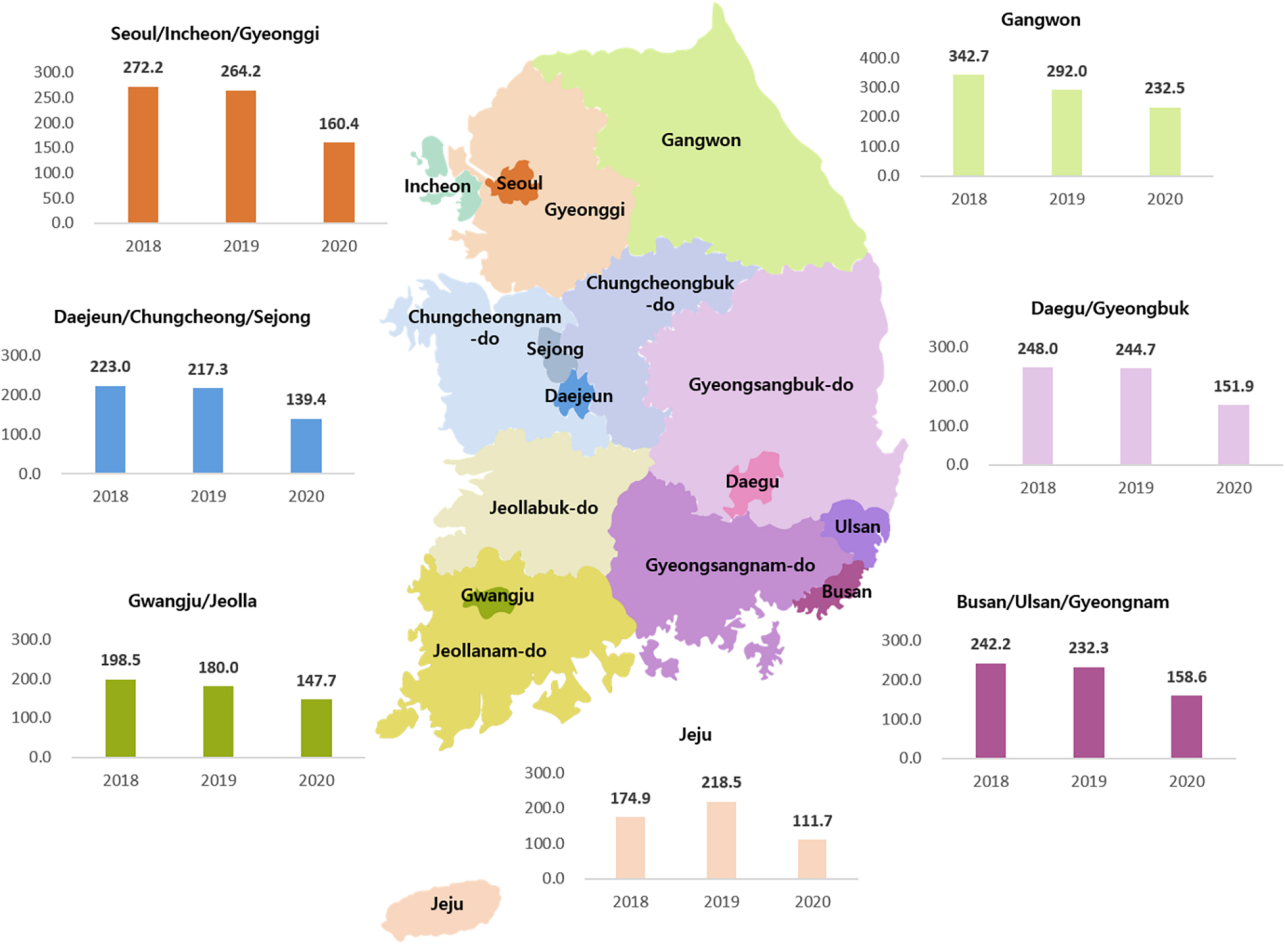
Yearly incidence of Kawasaki disease per 100,000 individuals younger than 5 years by region from 2018 to 2020.

In terms of KD incidence in localities during 2018–2020, Gangwon-do had the highest incidence whereas Jeju-do had the lowest (Figure 3). The Gangwon-do area has many mountains and is located at a high altitude. Jeju-do is surrounded by sea, is located at a low altitude, and has a hot and humid climate. After the start of the pandemic, the geographical differences remained constant, with Gangwon-do experiencing a slight decrease and Jeju-do experiencing the largest decrease in KD incidence owing to a high incidence rate in 2019. Compared with the incidence in the previous year, there was a minor decrease in the KD incidence in Gangwon-do and the largest decrease in Jeju-do, likely because of a high incidence rate in 2019.

## 4 Discussion

In this investigation, we explored the incidence of KD during the COVID-19 pandemic, utilizing NHIS data spanning from 2012 to 2020. An initial concern prior to this study was the emergence of Multisystem Inflammatory Syndrome in Children (MIS-C), which presents with symptoms similar to KD during the pandemic period. We considered the possibility of overlap in the data sets; however, in 2020, there were only three confirmed cases of MIS-C in South Korea. An expert panel reviewed these cases using strict diagnostic criteria to distinguish MIS-C from other conditions, including KD. The diagnosis of MIS-C was controlled using stringent criteria through a national control system, significantly minimizing its impact on our findings (18, 19). The observed decrease in KD incidence in 2020 could be attributed to pandemic-induced behavioral modifications, such as enhanced hand hygiene and social distancing practices, which may have potentially diminished the prevalence of agents that trigger KD. Several studies from various countries in East Asia and North America, including the Republic of Korea, Japan, and the United States, have reported a noticeable reduction in KD incidence following the onset of the pandemic in comparison to the incidence rates before the pandemic (20). Several factors, including reduced respiratory virus transmission, lower air pollution, and avoidance of healthcare facilities, may have contributed to the observed decrease in KD incidence during the pandemic (6, 12, 21, 22). In particular, reports associating KD with infectious triggers can provide insights that complement our study. Reports linking KD to infections such as H1N1 influenza prior to the COVID-19 pandemic, as well as investigations into KD-specific RNA viruses, serve as evidence for the presence of infectious triggers (23, 24). A recent study examining the relationship between KD and respiratory infections during the pandemic highlighted the potential role of infectious triggers in KD development. This finding supports previous hypotheses regarding the etiology of KD and underscores the need for continued investigation into the link between infectious agents and the onset of this condition (22). Furthermore, we found that the previously observed biphasic seasonality of KD incidence disappeared during this period, which potentially supports the idea that seasonal respiratory infections are among the triggers of the disease.

An additional finding was the marked shift in the age group majorly affected by KD. Specifically, we observed that the incidence rate in children aged <1 year was high compared with that in the other age groups, with a significant increase from the values in the previous years. This result is consistent with those of previous studies conducted in the United States and Japan and domestic multicenter studies (11, 25). As expected, the social behavior of children under 1 year of age is unlikely to have changed significantly between the pre-pandemic and pandemic periods. This is also the age at which it is more difficult to wear a mask and maintain it consistently, and while there is no prior research to support a mechanism, it is possible that they were spending more time at home with many family members than before the pandemic, leading us to hypothesize that exposure to more densely populated environments may also play a role. However, it is difficult to confirm this conclusively due to the limitations of objective data collection and analysis.

Another possible reason for the decrease in KD during the pandemic is the fear of parents of patients visiting the hospital (21). Thus, some expressed concerns about missing treatment due to a delay in diagnosis. Nevertheless, our data analysis indicated that treatment outcomes did not significantly differ in cases of IVIG-refractory KD or hospitalization periods between the pre-COVID-19 and COVID-19 pandemic periods, alleviating these concerns.

Extensive analyses of large datasets have revealed varied geographic patterns in the incidence of KD. National surveys conducted before 2010 indicated significant regional variations in the incidence rates, with areas such as Gangwon Province experiencing high incidence and others, such as Jeju-do, demonstrating low incidence rates (26). Subsequently, a recent study of the regional incidence of KD using HIRA data showed that the regions with the highest incidence of KD are the central and northern regions of South Korea, with a noticeable shift of the peak from the central region to the northeast and east coast between 2011 and 2012 and 2016–2017 (15). In contrast to our study, their data collection relied on a single diagnosis of KD. Consequently, the raw data are not directly comparable to ours. Furthermore, while it does not specify an exact incidence rate for Jeju Island, the presented figures suggest it to be among the lowest. Our 2018 regional data indicate that Jeju Island has the lowest incidence rate, aligning with these findings, albeit with interpretative limitations. A reduction in human-to-human contact owing to the pandemic serves as a potential explanation for this regional variation. Considering that Jeju Island is a popular tourist destination, with a larger floating population, and given the significant changes in tourists over the COVID pandemic period, this dynamic is likely to have played a large role in the regional disparities in KD incidence. The tourism statistics managed by the Jeju-do special self-governing province tourism association show the sharp changes in tourists over this period (Supplementary Appendix Table S4). We also further discussed the reasons for the regional disparities and reviewed the data, noting that environmental factors, such as geography, precipitation, and temperature changes, may also play an important role. Gangwon Province has the highest average altitude compared to the rest of South Korea and has a low population density and mountainous terrain with low temperatures, while Jeju Island has a hot and humid climate at lower latitudes. Previous studies from Japan and Taiwan suggest that factors such as high precipitation, low temperature, high population density, and high latitude correlate with the KD incidence (27–29). Another epidemiological study in tropical regions close to the equator reported higher KD incidence during the rainy season (30). The mentioned factors, such as precipitation, temperature, population density, and high latitude, on their own, cannot define a uniform cause for the regional disparities observed in KD incidence. Thus, further extensive studies are warranted to acquire more uniform data, facilitating a comprehensive analysis. The limitations of this study are attributable to strong privacy concerns. We had limited access to personally identifiable data and were only able to obtain basic demographic information. As a result, the depth of our analysis was limited, and we were unable to access data pertaining to more detailed clinical features, key echo findings, laboratory results, or other data. This prevented us from conducting a more in-depth study of the phenomenon, as opposed to exploring the underlying mechanisms, and we could only speculate and rely on previous studies for confirmation.

Despite these limitations, the strength of this study lies in its use of large-scale medical insurance data, which enhances its representativeness and objectivity, and validates the results of previous studies, thereby enhancing their credibility. Another strength is that it sheds light on additional clues to the etiology of KD, revealing a clear decrease in KD incidence during the COVID-19 pandemic, an increase in the percentage of affected children under the age of 1 year, and changes in regional incidence rates. These results provide valuable insights for future studies aimed at elucidating the underlying mechanisms of KD and highlight the urgent need for additional research to fully understand the multifactorial contributions to the observed changes in KD incidence. The importance of continued investigation into this area cannot be overstated, as the quest to uncover the cause of KD remains a priority in the field.

In South Korea, the incidence data of KD have previously been reported in articles, covering the period from 2012 to 2017 (31, 32). Upon reviewing these data, we found that the incidence rates recorded in our study differed from those previously reported. Previous studies, which were regularly conducted and published domestically through nationwide surveys, depended on data collected directly from various institutions via mail. This method presents the limitation that certain diagnosed cases might have been overlooked due to incomplete responses, potentially leading to the discrepancies observed between our data, data sourced from the HIRA, and the figures previously published. Even when using the same HIRA data warehouse to extract and analyze the same raw data, discrepancies were noted (15, 16). One study extracted KD cases using only diagnostic codes for data collection (15), which could result in numbers being overestimated since the diagnosis of KD can be included even in uncertain cases. Another study collected data using diagnostic codes along with codes for medication treatment; however, it was unclear whether the fulfillment of medication treatment conditions was a mandatory criterion or whether the treatment was administered (16). Thus, we believed that collecting and analyzing data on cases accurately diagnosed with KD would more closely reflect the actual incidence rates. Our data collection utilized South Korea's insurance reimbursement system, applying the guidelines published by the American Heart Association in 2017 for IVIG use, which covers the cost of IVIG under national insurance. We used conditions that required a diagnosis of KD along with the drug code for IVIG and strengthened the criteria by including only hospitalized patients to ensure the actual use of IVIG. Consequently, our study demonstrated incidence rates that were intermediate between those reported in the two prior studies using the NHIS database, and slightly higher than those reported by national epidemiological surveys (15, 16, 31, 32). This suggests that our research methodology, which employed more objective criteria for data collection, likely presented results closer to the realistic figures of KD incidence.

## 5 Conclusions

Our comprehensive big data study indicated a noticeable decline in KD cases during the COVID-19 pandemic, a rise in the proportion of affected infants below 1 year of age, and variations of incidence rate in regions with diverse climates and geographic settings. These findings hold crucial implications as valuable leads in unraveling the intricate mechanisms underlying KD.

## Data Availability

Data used in this study are available from the authors upon reasonable request, and with the permission of HIRA bigdata Open portal: https://opendata.hira.or.kr/home.do.
