# Supplementary material for: Decreased incidence of Kawasaki disease in South Korea during the SARS-CoV-2 pandemic
Source: Front Pediatr. 2024 Apr 3;12:1307931. doi: 10.3389/fped.2024.1307931 (PMC11021727; doi:10.3389/fped.2024.1307931)
Supplement: Supplementary file 1 [file Table1.docx]

**Supplementary material**

**Table S1. Discrepancies in incidence among various studies**

It is indeed pertinent to acknowledge that even when utilizing data from the same source, such as the National Health Insurance Service (NHIS), variations in the number of cases can arise depending on the search criteria employed. This table shows the number of Kawasaki disease(KD) cases obtained from two studies using NHIS data for each of the same years from 2012 to 2017 and a study conducted through a nationwide survey.

| **Articles PMID  (KD diagnostic criteria for data collection)** | **2012** | **2013** | **2014** | **2015** | **2016** | **2017** |
| --- | --- | --- | --- | --- | --- | --- |
| **PMID: 36760687**  **(**Only ICD-10-CM code* : M303**)** | 6,977 | 8,097 | 7,511 | 7,442 | 8,746 | 7,787 |
| **PMID: 35129593**  **(**M303 code and the use of various drugs, including IVIG**)** | 4,941 | 5,469 | 5,679 | 5,475 | 5,255 | 4,837 |
| **Nationwide survey**  **PMID: 27997519**  **PMID: 33075217**  (KD Diagnosis from respective hospitals) | 4,584 | 5,181 | 5,144 | 5,235 | 4,952 | 4,577 |
| **Our study reports**  **(**Satisfied both M303 code and the primary use of IVIG**)** | 5,363 | 5,691 | 5,907 | 5,693 | 5,422 | 4,995 |

* ICD-10-CM code: International Classification of Diseases, Tenth Revision, Clinical Modification code

**Table S2. Comparison of KD incidence per 100,000 between our data and that of previous nationwide surveys**

|  | **KD incidence per 100,000**  **Previous Nationwide survey studies’ data** | **KD incidence per 100,000**  **Our study data** |
| --- | --- | --- |
| **2012** | 190.2 | 201.7 |
| **2013** | 210.4 | 224 |
| **2014** | 217.2 | 236.9 |
| **2015** | 202.2 | 231.2 |
| **2016** | 197.1 | 222 |
| **2017** | 191.0 | 217.3 |
| **2018** |  | 238.9 |
| **2019** |  | 230 |
| **2020** |  | 141.2 |

**Table S3. Prescription history of collected medications**

| **Medication** | **Prescription code** |
| --- | --- |
| Intravenous immunoglobulin | 169930BIJ, 169931BIJ, 169932BIJ, 169933BIJ, 169935BIJ, 169936BIJ, 169937BIJ, 169938BIJ, 169939BIJ, 169940BIJ |
| Aspirin | 111001ACE, 111001ATB, 111001ATE, 111002ATE, 111003ACE, 111003ATE, 110701ATB, 110702ATB, 110801ATB, 110802ATB, 256800ATB, 667500ACE |
| Corticosteroids | 217001ATB, 217034ASY, 217035ASY, 193601BIJ, 193603BIJ, 190304BIJ |
| Infliximab | 383501BIJ, 383502BIJ |
| Clopidogrel | 136901ATB, 517900ACE, 517900ATE |
| Anticoagulation drugs** | 249103ATB, 209105ATB, 152130BIJ, 152131BIJ, 152132BIJ, 152133BIJ, 152134BIJ, 168636BIJ, 168630BIJ, 168632BIJ, 168637BIJ, 168638BIJ, 168631BIJ |

**unfractionated heparin, low-molecular heparin, and vitamin K

**Table S4. Jeju Island tourist arrivals from 2017 to 2020**

| **Year** | **Total Visitors** | **Domestic Visitors** | **International Visitors** | **Year-over-Year Change (%)*** |
| --- | --- | --- | --- | --- |
| **2017** | 14,753,236 | 13,522,632 | 1,230,604 | -6.9 |
| **2018** | 14,313,961 | 13,089,129 | 1,224,832 | -3 |
| **2019** | 15,286,136 | 13,560,004 | 1,726,132 | +6.8 |
| **2020** | 10,236,445 | 10,023,678 | 212,767 | -33 |

*The "Year-over-Year Change" column represents the percentage change in the total number of visitors compared to the previous year

The data used in this table were sourced from the tourism statistics managed by the Jeju Special Self-Governing Province Tourism Association. The data can be accessed at the following website: http://www.visitjeju.or.kr/web/bbs/bbsList.do?bbsId=TOURSTAT
